# Epidemiology and microbiology of recurrent UTI in women in the community in Oxfordshire, UK

**DOI:** 10.1093/jacamr/dlad156

**Published:** 2024-01-10

**Authors:** Maria D L A Vazquez-Montes, Thomas R Fanshawe, Nicole Stoesser, A Sarah Walker, Christopher Butler, Gail Hayward

**Affiliations:** Nuffield Department of Primary Health Care Sciences, University of Oxford, Radcliffe Primary Care Building, Radcliffe Observatory Quarter, Woodstock Road, Oxford OX2 6GG, UK; Nuffield Department of Primary Health Care Sciences, University of Oxford, Radcliffe Primary Care Building, Radcliffe Observatory Quarter, Woodstock Road, Oxford OX2 6GG, UK; Nuffield Department of Medicine, University of Oxford, John Radcliffe Hospital, Oxford OX3 9DU, UK; Modernising Medical Microbiology Consortium, University of Oxford, Experimental Medicine Division, John Radcliffe Hospital, Headley Way, Headington, Oxford OX3 9DU, UK; Nuffield Department of Medicine, University of Oxford, John Radcliffe Hospital, Oxford OX3 9DU, UK; Nuffield Department of Primary Health Care Sciences, University of Oxford, Radcliffe Primary Care Building, Radcliffe Observatory Quarter, Woodstock Road, Oxford OX2 6GG, UK; Nuffield Department of Primary Health Care Sciences, University of Oxford, Radcliffe Primary Care Building, Radcliffe Observatory Quarter, Woodstock Road, Oxford OX2 6GG, UK

## Abstract

**Background:**

Recurrent urinary tract infection (rUTI) contributes to significant morbidity and antibiotic usage.

**Objectives:**

To characterize the age of women experiencing rUTI, the microbiology of rUTIs, and the risk of further rUTIs in Oxfordshire, UK.

**Patients and methods:**

We retrospectively analysed de-identified linked microbiology and hospital admissions data (Infections in Oxfordshire Research Database), between 2008 and 2019, including positive urine cultures from women aged ≥16 years in community settings. We defined rUTI as ≥2 positive urine cultures within 6 months or ≥3 within 12 months.

**Results:**

Of 201 927 women with urine culture performed, 84 809 (42%) had ≥1 positive culture, and 15 617 (18%) of these experienced ≥1 rUTI over a median (IQR) follow-up of 6 (3–9) years. Women with rUTI were 17.0 (95% CI: 16.3–17.7) years older on average. rUTI was commonest (6204; 40%) in those aged 70–89 years. Post-rUTI, the risk of further UTI within 6 months was 29.4% (95% CI: 28.7–30.2). *Escherichia coli* was detected in 65% of positive cultures. Among rUTIs where the index UTI was *E. coli* associated, the second UTI was also *E. coli* associated in 81% of cases.

**Conclusions:**

rUTIs represent a substantial healthcare burden, particularly in women >60 years. One-third of women experiencing rUTI have a further microbiologically confirmed UTI within 6 months.

## Introduction

Urinary tract infection (UTI) is the commonest bacterial infection managed in primary care.^1,2^ UTI is much more common in women than in men, with 50% lifetime incidence in women.^3^ Recurrent UTI (rUTI), defined as ≥2 UTI episodes in 6 months or ≥3 in a year,^3^ is associated with significant morbidity and reduction in quality of life.^4^ Women report impact on their sexual relationships, ability to work and travel, and systemic physical symptoms in addition to painful and frequent urination.^5^ Long-term antibiotics are the mainstay of prophylaxis for rUTI,^6,7^ but there is limited evidence for effectiveness beyond the period of prophylaxis,^8^ and prophylaxis may increase the risk of developing antimicrobial resistance.^9^

It is important to understand the epidemiology and characteristics of women with rUTI to appropriately target current prophylactic options and include the right patients in trials of novel therapies for this debilitating condition. However, rUTI is poorly characterized in the UK population and worldwide, in part because most databases of primary care contacts for UTI do not include detailed microbiological outcome data. Estimates of the proportion of women experiencing recurrent infection vary from 3% to 50%.^2,10,11^ However, the studies providing these estimates often do not report microbiological confirmation of infection, the populations included vary in age and demographics, and some use varying recurrence timelines.

To address this evidence gap, we used anonymized linked microbiological data and hospital electronic health records from the UK region of Oxfordshire, covering ∼700 000 people, to describe the population of women experiencing rUTI in community settings, the microbiology of these infections, and the risk of subsequent UTI episodes.

## Patients and methods

The Infections in Oxfordshire Research Database (IORD, https://oxfordbrc.nihr.ac.uk/research-themes/modernising-medical-microbiology-and-big-infection-diagnostics/infections-in-oxfordshire-research-database-iord/) is an NIHR Oxford Biomedical Research Centre-supported de-identified electronic database containing linked microbiology results from specimens taken in primary and secondary care (including urine culture results) and tested at the Oxford University Hospitals NHS Foundation Trust (the only provider of acute care in the region), patient demographics and clinical records.

### Ethics

IORD has approval from the South Central Research Ethics Committee (19SC/0403) and Confidentiality Advisory Group of the Health Research Authority (19CAG0144) for research without individual patient consent.

### Inclusion criteria

Participants were female and aged 16–105 years who had at least one urine sample sent for culture from healthcare settings in the region between April 2008 and March 2019. We included urine cultures requested from primary care settings and cultures requested within 48 h after an inpatient admission to hospital, provided the participant did not have an inpatient hospital admission in the previous 28 days. The remaining cultures were considered hospital onset and excluded. We also excluded cultures without microbiology results, where the test failed or was explicitly requested for antenatal screening, and catheter specimens.

For quality control, we excluded a small number of records for the following reasons: evidence of record mislinkage (e.g. a linked date of death prior to the study period, culture collection date listed as >48 h after the patient’s date of death); and test results dated >24 h before the recorded specimen collection date.

There were no available data on symptoms or urine dipstick results in this dataset. As the urine samples analysed in this study were requested by a clinician, we expect that the majority were from women who were symptomatic, leading the clinician to require a diagnostic test to confirm or rule out infection, especially given the resources required to collect the samples, send them to the laboratory and process the tests, and given the absence of any recommendations for testing for asymptomatic bacteriuria in the UK. A positive urine culture, in addition to relevant symptoms, would generally be considered as sufficient to meet the current diagnostic criteria for UTI in primary care, and so in this study we have used bacteriuria as a proxy for UTI.

### Definitions of UTI and rUTI

In the primary analysis, an episode of UTI was defined by a positive culture of a known uropathogen (defined in the laboratory as pure or predominant growth at ≥10^4^ cfu/mL). We defined a new subsequent UTI episode by a positive culture occurring 28 days or more from the index date of a previous UTI episode.^12^ Sensitivity analysis defined any culture result (whether positive, mixed, equivocal or negative) as indicating symptoms attributable to a possible UTI, since up to two-thirds of women presenting to primary care with UTI symptoms do not have a positive urine culture,^13^ and a clinician’s request for a urine culture indicates clinical suspicion of UTI.^7,14^ A further sensitivity analysis allowed a shorter time to define a subsequent episode of ≥14 days. We applied the current definition of rUTI ( ≥ 2 UTIs in 6 months or ≥3 in 12 months),^3^ regarding a period of 6 months without a UTI as ending the rUTI event. The index rUTI for a participant was defined as the first occasion the rUTI definition was met during the study period.

### Microbiology

Microbiological characteristics of rUTI were described and compared using the cohort from 1 June 2013 onwards, following significant changes to laboratory species identification and susceptibility testing in 2013 with the introduction of automated broth microdilution methods. Microbiological results were grouped into seven categories (Table S1, available as Supplementary data at *JAC-AMR* Online).

For each of *Klebsiella* spp., *Proteus* spp., *Escherichia coli*, *Enterococcus faecalis* and *Enterococcus* faecium (with the two *Enterococcus* species reported separately because they have different resistance phenotypes), we calculated the proportion resistant to commonly prescribed antibiotics for treating UTI in primary care (amoxicillin, amoxicillin/clavulanate (co-amoxiclav), cefalexin, ciprofloxacin, fosfomycin, nitrofurantoin, pivmecillinam and trimethoprim). These proportions were calculated at index UTI and at index rUTI.

### Statistical analysis

Summary statistics were presented using median (IQR) or as percentages, as appropriate for the data type. The age profile of participants was summarized in age groups (16–49, 50–64, 65+ years) and by age in decades. Baseline age was defined as age at the index UTI of the first rUTI where experienced, or at the first UTI after 2008 otherwise, and compared using median quantile regression (*qreg* command in Stata).

For participants with at least one rUTI, we calculated Kaplan–Meier estimates for the risk of experiencing one, two and three subsequent UTIs within 6 and 12 months following the index rUTI. For participants with no rUTI, similar quantities were calculated based on the date of the first (index) UTI.

A chi-squared test was used to assess the association between bacterial species and UTI recurrence. The effect of species and age group were evaluated using a multinomial logistic regression (*mlogit* command in Stata) with species as the dependent variable and *E.coli*, experiencing any rUTI, and age 30–39 years as reference categories. We used the Bayesian information criterion (BIC) to test whether the age effect varied by recurrence group, by including an interaction between these variables. We reported relative risk ratios (RRRs).

Reinfection with the same bacterial species was summarized by cross-tabulating the bacterial species in the first and subsequent UTI of all rUTI episodes experienced by rUTI women. Similarly, species in pairs of consecutive UTI episodes across women with ≥2 UTIs who did not experience any rUTI in the study period were cross-tabulated.

We calculated the proportion of *E.coli-*related index UTIs resistant to ciprofloxacin out of all *E.coli-*related index UTIs tested for ciprofloxacin susceptibility. We repeated this for each bacterial species and each of the antibiotics considered in this study, for women who experienced ≥1 rUTI episode and those who did not. We compared the proportions in the two recurrence groups using a Z-test for proportions.

Analyses were performed using Stata 16.1. Results were reported with 95% CIs.

## Results

### Urine cultures

**Figure 1.**
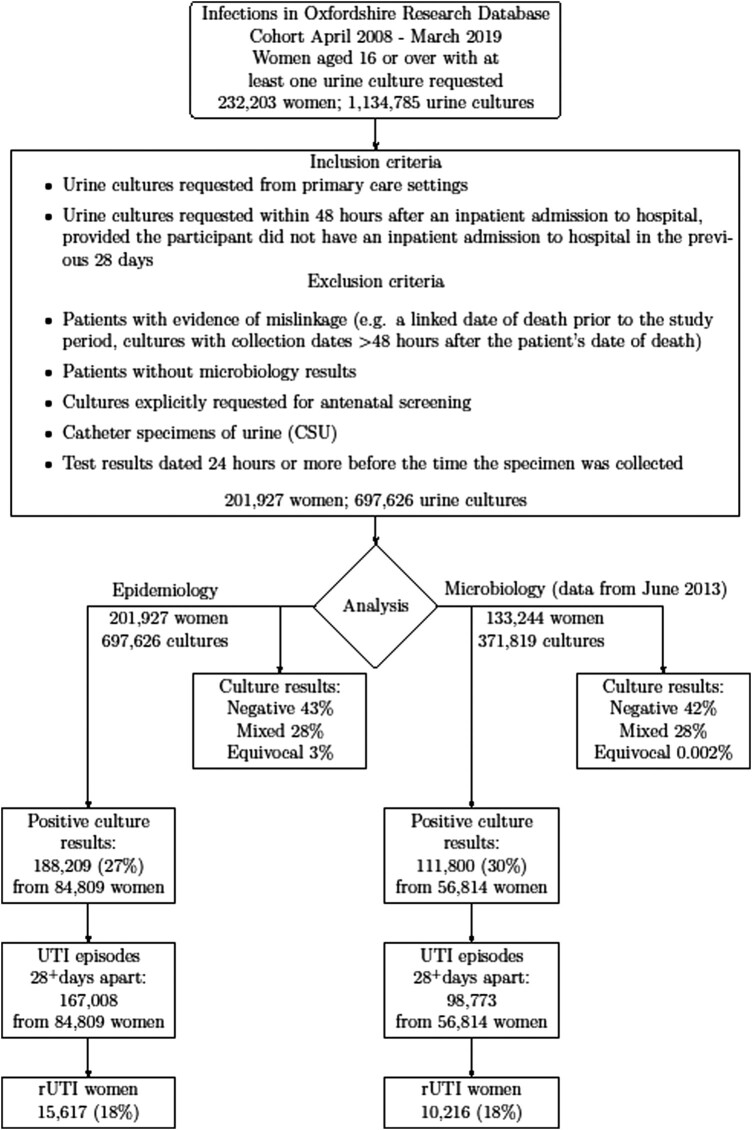
Data management flow chart for primary analysis.

We included 697 626 urine culture results performed on samples from 201 927 women taken between April 2008 and March 2019 (Figure 1), with median (IQR) follow-up of 6 (3–9) years per person. Of these, 27% were culture positive, 28% showed mixed growth, 3% equivocal growth and 43% were negative. Fifty-eight percent of women (118 095) had more than one culture result (median 3, IQR 2–6 per woman). A much higher percentage of urine samples taken from participants aged 30–39 years were negative (59%; 22 293/37 985), possibly because they were taken for antenatal screening but not coded as such (Figure S1).

Forty-two percent of women (84 809) had at least one positive culture during the study period, with less than half of these (34 473; 41%) having two or more positive cultures (median 3, IQR 2–4 per woman). After excluding multiple positive cultures recorded within 28 days, there were 167 008 UTI episodes in total. Table S2 presents the number of women with one or two or more UTI episodes during the study period, and shows the impact of using a window of 14 or 28 days to define UTI episodes.

### Epidemiology of rUTI

Of the 167 008 UTI episodes, 54 512 (33%) were part of rUTIs experienced by a total of 15 617/84 809 (18%) women who had at least one positive culture, with 20 744 distinct rUTI events (≥2 UTIs in 6 months or ≥3 in 12 months) overall (range 1–7 per woman), and most rUTI events consisting of only two UTIs (14 135; 68%). Seventy-eight percent of women (12 184) experienced a single rUTI event, 2305 (15%) two rUTIs separated by a period longer than 6 months without any UTI, and 1128 (7%) three or more rUTIs over the study period.

**Figure 2.**
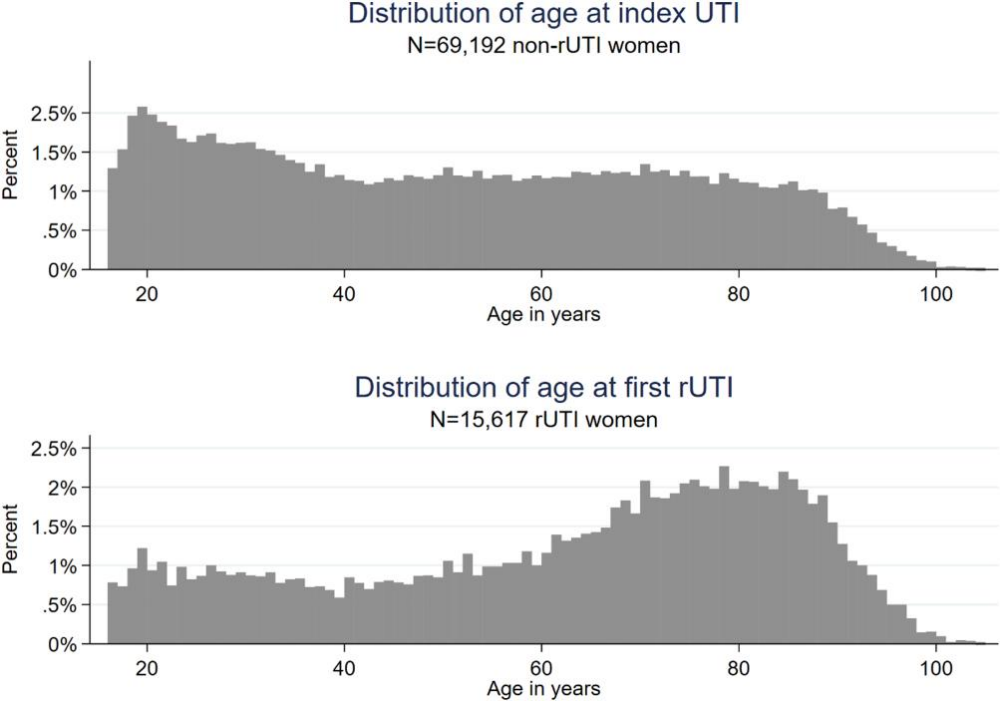
Histograms of the distribution of age at index UTI and index rUTI for women with positive cultures collected between 2008 and 2019.

Women who experienced rUTIs had a median age of 17.0 (95% CI: 16.3–17.7) years greater than those who had an index UTI but never had a rUTI in the study period [median (IQR) 67 (45–80) versus 50 (30–70) years] (Figure 2). Those with rUTIs were most commonly aged 70–79 or 80–89 [3140 (20%) and 3064 (20%), respectively], whereas the most common age group of those with UTI but not meeting the criteria for UTI recurrence was 20–29 years (11 953; 17%) (Table 1).

**Table 1.** Distribution of age at index UTI and index rUTI for women with positive cultures between 2008 and 2019

Age	Age at index UTI non-rUTI women *N* = 69 192		Age at index rUTI rUTI women *N* = 15 617	
	50 (30–70)		67 (45–80)	
	*n*	%	*n*	%
Age group (years), median IQR				
* *16–49	34 265	50	4467	29
* *50–64	12 454	18	2625	17
* *65+	22 473	32	8525	55
Age in decades (years), median IQR				
* *16–19	4754	7	576	4
* *20–29	11 953	17	1420	9
* *30–39	9606	14	1217	8
* *40–49	7952	11	1254	8
* *50–59	8303	12	1592	10
* *60–69	8405	12	2304	15
* *70–79	8421	12	3140	20
* *80–89	7119	10	3064	20
* *≥90	2679	4	1050	7

**Figure 3.**
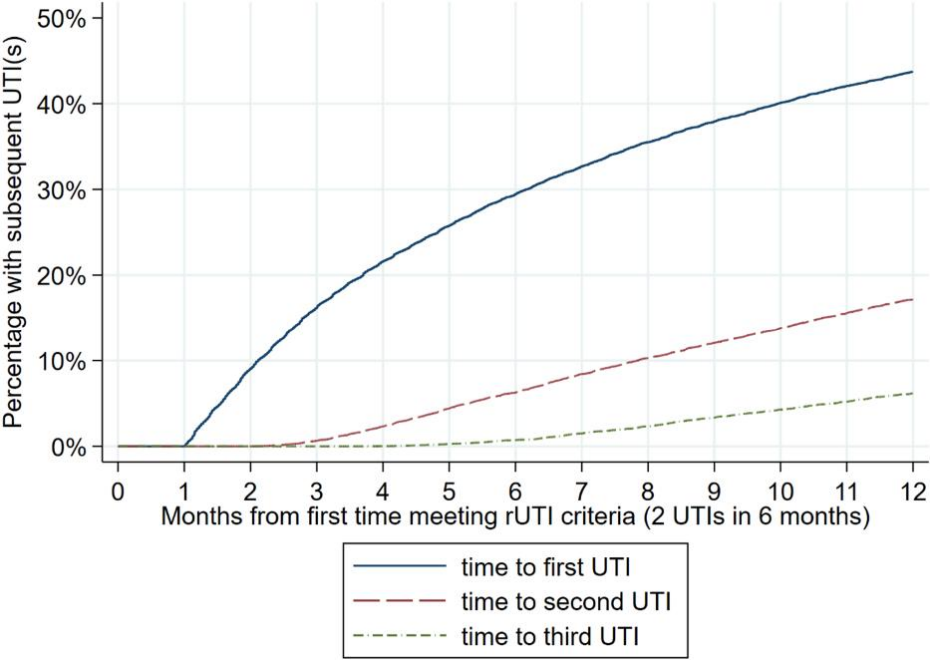
Time to first, second and third subsequent UTI from the first time the rUTI criteria are met for an index rUTI event, in the 15 617 women who experienced at least one rUTI episode.

The risk of having a subsequent UTI after an index rUTI event was 29.4% (95% CI: 28.7–30.2) within 6 months and 43.7% (95% CI: 42.9–44.6) within 12 months (Figure 3). The risk of having two further UTIs within these periods was 6.3% (95% CI: 5.9–6.7) and 17.2% (95% CI: 16.6–17.8) respectively; and the risk of having three further UTIs was 0.7% (95% CI: 0.6–0.9) and 6.2% (95% CI: 5.8–6.6) respectively. Kaplan–Meier plots stratified by age followed a similar pattern, with risk increasing with age (Figure S2).

Results were similar whether a minimum window of 14 or 28 days was used to define UTI episodes (Table S3). Including all urinary cultures (whether positive or not) as indicators of UTI, 64 260 (32%) women met the rUTI definition during the study period.

### Microbiology

Between June 2013 and March 2019, there were 111 800 positive cultures from 56 814 women (Figure 1), of which 73 214 (65%) grew *E. coli* and 4037 ESBL-*E. coli* (4%) (Table 2). Percentages of other species were much smaller [*Klebsiella* spp.: 7590 (7%); ESBL-*Klebsiella* spp.: 404 (0.4%); *Enterococcus* spp.: 7252 (6%); *Proteus* spp.: 3388 (3%)]. Pathogen distribution was broadly similar across age groups, with 7% (95% CI: 7–8) more *Enterococcus* spp. in the 30–39 age group and 4% (95% CI: 4–5) more *Klebsiella* spp. in the 80–89 age group.

**Table 2.** Distribution of the main bacterial species, overall and within age groups

	Age groups (years)																			
	16–19		20–29		30–39		40–49		50–59		60–69		70–79		80–89		≥90		Total	
	*n*	%	*n*	%	*n*	%	*n*	%	*n*	%	*n*	%	*n*	%	*n*	%	*n*	%	*n*	%
*E. coli*	2603	68	7691	64	6326	59	6971	70	8851	70	10 525	68	14 275	66	11 892	62	4080	62	73 214	65
ESBL-*E. coli*	99	3	356	3	305	3	329	3	431	3	596	4	886	4	757	4	278	4	4037	4
*Enterococcus* spp.	178	5	963	8	1380	13	646	6	693	5	887	6	1167	5	1038	5	300	5	7252	6
*Klebsiella* spp.	104	3	423	4	407	4	442	4	690	5	1104	7	1862	9	1964	10	594	9	7590	7
ESBL-*Klebsiella*	6	0	16	0	26	0	19	0	48	0	60	0	88	0	98	1	43	1	404	0
*Proteus* spp.	111	3	267	2	189	2	150	2	274	2	465	3	753	3	850	4	329	5	3388	3
Other	754	20	2220	19	2008	19	1406	14	1661	13	1899	12	2534	12	2492	13	941	14	15 915	14

The baseline distribution of bacterial species was statistically significantly (*P* < 0.001) different for those who experienced ≥1 rUTI and those who did not in the study period, at the time of index UTI and index rUTI, although differences were small. Table S4 presents these proportions overall and by age. *E. coli* was the most commonly cultured pathogen in both groups [6921 (68%) in rUTI women; 30 915 (66%) in non-rUTI women]. All other species were rarer, for instance, *Klebsiella* spp. [774 (8%) in rUTI women; 2232 (5%) in non-rUTI women]. The effect of age on the distribution of organisms was not different between recurrence groups (BIC was smaller for the model without an interaction between age and recurrence group). RRRs for specific species and age combinations are shown in Table S5.

### Risk of recurrence with the same species

Among rUTI events, there was an indication that the first UTI in a sequence was disproportionately likely to be followed by a subsequent UTI with the same species. For example, among rUTI sequences where the index UTI was *E. coli* associated, 81% (6813/8377) also had an *E. coli*-associated UTI at the time of recurrence (Table S6). A similar pattern occurred for rarer species: for instance, 37% (156/426) of index *Proteus* spp. UTIs that formed part of a rUTI sequence were followed by a *Proteus* rUTI, even though *Proteus* was detected in only 3% of positive cultures overall. In the 8471 non-rUTI women who experienced another UTI outside the 6 month window defining recurrence, the pattern of repeat infection remained elevated but was slightly less pronounced (e.g. only 77% of *E. coli* UTIs were followed by another *E. coli* UTI in non-rUTI women with multiple infections) (Table S7).

### Antibiotic resistance

Information about antibiotic susceptibility phenotypes for cultured isolates was available for 109 177 (97.7%) positive cultures collected from June 2013 from 55 720 (98.1%) women in the study, and from 10 191 (99.8%) of those who experienced any rUTI. The availability of susceptibility testing for each pathogen–antibiotic combination was affected by EUCAST guidance, for example resulting in no data for resistance to pivmecillinam or fosfomycin in *Enterococcus* spp., low numbers for isolates tested for cases where intrinsic resistance is thought to be prevalent [e.g. cefalexin for *Enterococcus* spp. (109/2705 isolates tested)] and high prevalence of resistance reported for species–drug combinations where resistance is again thought to be intrinsic (e.g. *Klebsiella* spp. and amoxicillin). There was some evidence that a greater proportion of *E. coli* isolates were more resistant to several of the common antibiotics in index rUTIs, compared with index UTI in non-rUTI women, although these differences were small (Table 3).

**Table 3.** Percentage of bacterial species resistant to each of the most commonly prescribed antibiotics to treat UTI in index samples for those who experienced rUTIs and those who did not experience rUTIs in the study period

Bacterial species	Commonly prescribed antibiotics to treat UTI															
	CIP		AMC		NIT		TMP		AMX		LEX		FOF		PIV	
	$\frac{n}{total}$	%	$\frac{n}{total}$	%	$\frac{n}{total}$	%	$\frac{n}{total}$	%	$\frac{n}{total}$	%	$\frac{n}{total}$	%	$\frac{n}{total}$	%	$\frac{n}{total}$	%
*E. coli*																
rUTI	$\frac{504}{6742}$	7	$\frac{1807}{6746}$	27	$\frac{113}{6710}$	2	$\frac{1908}{6748}$	28	$\frac{2889}{6746}$	43	$\frac{833}{6327}$	13	$\frac{68}{6690}$	1	$\frac{419}{3960}$	11
Non-rUTI	$\frac{1602}{30790}$	5	$\frac{7216}{30733}$	23	$\frac{269}{30570}$	1	$\frac{7814}{30733}$	25	$\frac{12635}{30761}$	41	$\frac{3109}{28845}$	11	$\frac{266}{30502}$	1	$\frac{1494}{18525}$	8
Difference in % (95% CI)	2 (2–3)		3 (2–4)		0.8 (0.5–0.1)		3 (2–4)		2 (1–3)		2 (2–3)		0.1 (−0.1 to 0.4)		3 (2–3)	
*E. faecalis*																
rUTI	$\frac{267}{330}$	81	$\frac{0}{9}$	0	$\frac{0}{425}$	0	$\frac{424}{425}$	99.8	$\frac{0}{429}$	0	$\frac{12}{12}$	100	—	—	—	—
Non-rUTI	$\frac{1849}{2278}$	81	$\frac{3}{62}$	5	$\frac{10}{2865}$	0.4	$\frac{2840}{2848}$	99.7	$\frac{3}{2932}$	0.1	$\frac{81}{82}$	99	—	—	—	—
Difference in % (95% CI)	−0.3 (−4 to 4)		−5 (−9 to −0.4)		−0.3 (−0.5 to −0.2)		0.1 (−0.4 to 0.5)		−0.1 (−0.2 to −0.0)		1 (−0.8 to 3)		—		—	
*E. faecium*																
rUTI	$\frac{22}{22}$	100	$\frac{3}{3}$	100	$\frac{20}{25}$	80	$\frac{26}{26}$	100	$\frac{23}{25}$	92	$\frac{3}{3}$	100	—	—	—	—
Non-rUTI	$\frac{72}{75}$	96	$\frac{8}{9}$	89	$\frac{70}{100}$	70	$\frac{96}{98}$	98	$\frac{80}{100}$	80	$\frac{12}{12}$	100	—	—	—	—
Difference in % (95% CI)	4 (0.3–8)		11 (−6 to 28)		10 (−5 to 25)		2 (−0.3 to 4)		12 (1–23)		—					
*Klebsiella* spp.																
rUTI	$\frac{3}{157}$	2	$\frac{25}{157}$	16	$\frac{28}{155}$	18	$\frac{16}{158}$	10	$\frac{158}{159}$	99	$\frac{52}{149}$	35	$\frac{36}{156}$	23	0/3	0
Non-rUTI	$\frac{6}{422}$	1	$\frac{74}{423}$	17	$\frac{55}{420}$	13	$\frac{27}{423}$	6	$\frac{419}{425}$	99	$\frac{134}{401}$	33	$\frac{94}{423}$	22	0/1	0
Difference in % (95% CI)	0.5 (−2 to 3)		−2 (−7 to 4)		5 (−1 to 11)		4 (−1 to 8)		1 (−1 to 2)		1 (−6 to 9)		1 (−6 to 7)		—	
*Proteus* spp.																
rUTI	$\frac{2}{26}$	8	$\frac{2}{26}$	8	$\frac{26}{26}$	100	$\frac{13}{26}$	50	$\frac{19}{26}$	73	$\frac{17}{26}$	65	$\frac{3}{26}$	12	—	—
Non-rUTI	$\frac{1}{90}$	1	$\frac{14}{90}$	16	$\frac{87}{88}$	99	$\frac{25}{90}$	28	$\frac{57}{90}$	63	$\frac{44}{85}$	52	$\frac{13}{89}$	15	$\frac{2}{2}$	100
Difference in % (95% CI)	7 (−2 to 15)		−8 (−19 to 3)		1 (−1 to 3)		22 (4 to 40)		10 (−7 to 26)		14 (−4 to 31)		−3 (−15 to 9)		—	

There were 41 682/55 720 (75%) participants with susceptibility tests at index UTI; 7451/10 216 (73%) participants with susceptibility tests at index rUTI; total = total number of index UTIs tested for susceptibility to the particular antibiotic; CIP, ciprofloxacin; AMC, co-amoxiclav; NIT, nitrofurantoin; TMP, trimethoprim; AMX, amoxicillin; LEX, cefalexin; FOF, fosfomycin; PIV, pivmecillinam; some results could be due to ‘intrinsic resistance’ [EUCAST guidance (see Table S8)].^15,16^

## Discussion

### Summary of findings

This study demonstrates, to our knowledge for the first time, the burden of laboratory identified rUTI in women at a population and individual level, using a large UK electronic health record database that contained more than 200 000 women with at least one UTI episode. At the population level, we found at least 18% of women with any positive culture had one or more episodes of rUTI over a median follow-up of 6 years. The average number of women aged ≥16 years in Oxfordshire during 2008–19 was 274 300.^17^ This means that the estimated proportion of women in Oxfordshire who experienced microbiologically confirmed rUTI at some time during the study period is 6% (15 617/274 300), ignoring population changes.

The burden at the individual level was also significant: 29% of women who met criteria for rUTI experienced a further UTI episode within 6 months, and 6% had at least three further UTI episodes within a year. Women with rUTI tended to be older than those with non-recurrent UTI, with a median of 67 years at the time of first rUTI, compared with 50 for non-rUTI women.

The commonest uropathogen in both index UTIs and rUTIs was *E. coli*, found in approximately two-thirds of positive cultures. Repeated infection with the same uropathogen was typical for all bacterial species identified on culture and indicative of rUTI, although the extent of this varied by uropathogen. There was a tendency for *E. coli* samples to be more frequently resistant to common antibiotics in index rUTIs compared with index UTI in women without rUTI, although the size of this effect was small.

### Comparison with other literature

There are few studies that address the epidemiology of rUTI. A laboratory surveillance study in Canada^18^ with similar inclusion criteria to our study found that 10.5% of women with any microbiologically confirmed UTI experienced ≥3 positive cultures in a 2 year period. A review in 2014 estimated that the risk of UTI recurrence was 30%–50% per year in adult women,^2^ but it is unclear whether UTIs were microbiologically confirmed in this study and whether the estimate includes pregnant women and catheter-associated UTIs. A small prospective cohort of Finnish women recruited from primary care found a recurrence rate of 44.1% within 12 months after an index episode of cystitis caused by *E coli*, of whom 18.4% met the criteria for rUTI of ≥3 in a year.^19^

The frequencies of infecting organism found in this study are similar to a primary care-based cohort study of 1119 women in France^20^ and the larger ambulatory population in the Canadian study.^18^ Resistance to nitrofurantoin was lower in our study (1%–2%) than in the Canadian cohort (6%), but similar to rates reported in the French cohort.

Theories that have emerged from *in vitro* and mouse-model paradigms suggest that some rUTIs may represent re-emergence of infection from the bladder wall rather than reinfection^21^ from the perineum. We found that, even for the rarer species, reinfection with the same organism was more frequent. This might support the theory that at least some rUTIs result from re-emergence. Future isolate sequencing studies would facilitate evaluation of this.

### Strengths and limitations

This study is the first to use a UK dataset of longitudinal de-identified microbiology results linked with hospital admissions to explore the epidemiology of rUTI and provide novel insights regarding future risk of UTI in this population.

The study has a number of important limitations. This analysis is likely to underestimate the incidence of both UTI and rUTI, as it captures neither women who are prescribed antibiotics by their GP without a culture being sent, nor those who do not present to their GP. The latter is likely to have only a small impact as household surveys estimate that only 5%^10^ of women manage their UTI symptoms without contacting a healthcare professional. Scottish Intercollegiate Guidelines Network (SIGN) and NICE guidance^6,7^ suggest that women with rUTI should have a urine culture sent when consulting in primary care, but this is unlikely to have been applied in all cases, and could mean that the recurrent infection is captured but the index UTI is not, leading to underestimation of the rUTI burden. A second key limitation is that the lack of consultation data means that we could not distinguish between asymptomatic bacteriuria^22^ and UTI, meaning we have overestimated UTI episodes in the older adults included in this study. Although one might expect significantly fewer samples to be sent from asymptomatic cases, it has been common practice for frail people in care homes to have their urine dipstick tested and then sent for culture in the absence of a clear reason for a perceived acute decline in health status.^23^ However, in community populations the prevalence of asymptomatic bacteriuria in postmenopausal women remains very low,^24^ and therefore we believe that in the majority of cases bacteriuria will be reflective of UTI. The absence of clinical data also means that we are unable to distinguish between upper- and lower-tract UTI. We cannot be confident that our sample completely excludes catheter-associated UTI because we relied on coding of the clinical samples upon submission.

This study used data from Oxfordshire, and so findings may not be generalizable to other areas of the UK or international populations, especially given that the extent to which UTIs vary between different ethnic and socioeconomic groups is poorly understood. Previous analyses of this database have shown that the rates of sample submission vary considerably across different primary care practices.^25^

### Implications for practice/research

This study demonstrates that the main population who experience recurrent culture-positive UTIs are older women. This has implications for both practice and research. In practice, it could be important to consider vaginal oestrogen therapy to reduce the potential contribution of postmenopausal changes toward recurrent UTIs.^26^ In research, trials of medications for recurrent UTI should not place an upper age limit on recruitment in order to ensure interventions are being tested in the population most at risk. This further underlines the importance of better ascertaining what constitutes a true UTI as opposed to a microbiological definition of a positive urine culture. Especially in older people, the association between urine culture and clinical findings needs further clarification. The usual cut-off of 65 years used to distinguish between the best diagnostic approaches^6,7^ could be reconsidered in the context of these findings.

## Supplementary Material

dlad156_Supplementary_Data

## Supplementary data

Figures S1 and S2 and Tables S1 to S8 are available as Supplementary data at *JAC-AMR* Online.
