# Supplementary material for: Epidemiology and microbiology of recurrent UTI in women in the community in Oxfordshire, UK
Source: JAC Antimicrob Resist. 2024 Jan 10;6(1):dlad156. doi: 10.1093/jacamr/dlad156 (PMC10781434; doi:10.1093/jacamr/dlad156)
Supplement: dlad156_Supplementary_Data [file dlad156_supplementary_data.docx]

**Supplementary material**

**FIGURES**

Figure S1: Age at index culture in the whole cohort, by culture result


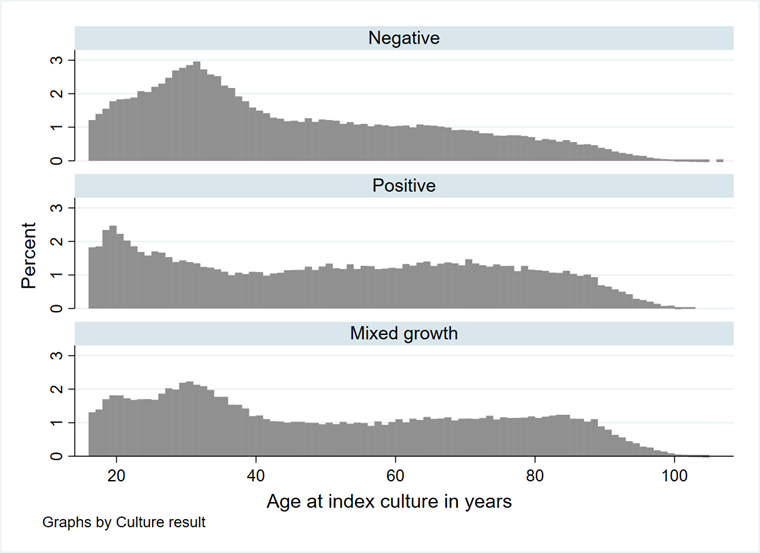


Figure S2: Time to subsequent UTIs by age group. Time to a) first, b) second and c) third subsequent UTI from the first time the rUTI criteria is met for an index rUTI event, stratified by age, in the 15,617 women who experienced at least one rUTI episode.

| 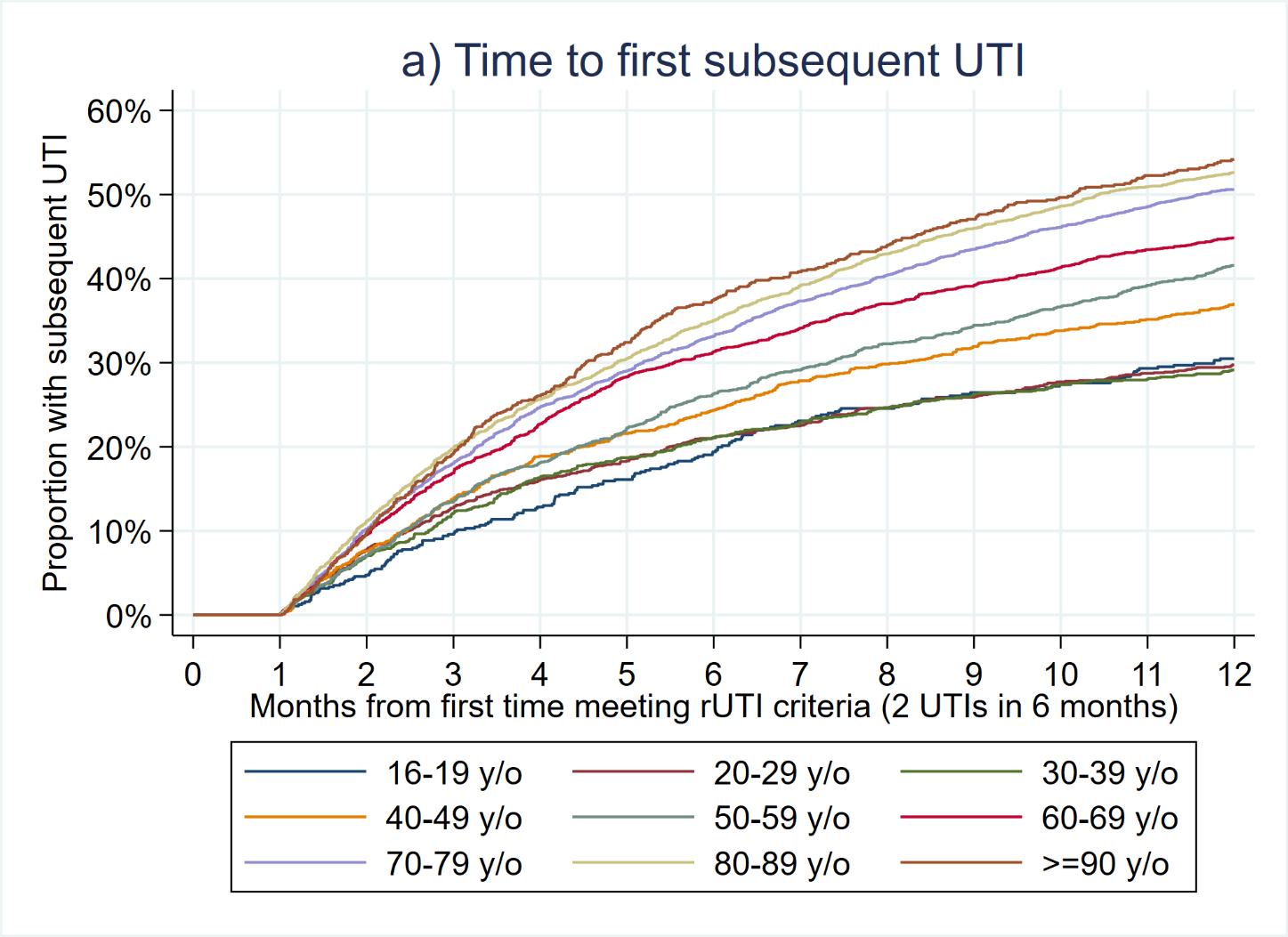 |
| --- |
| 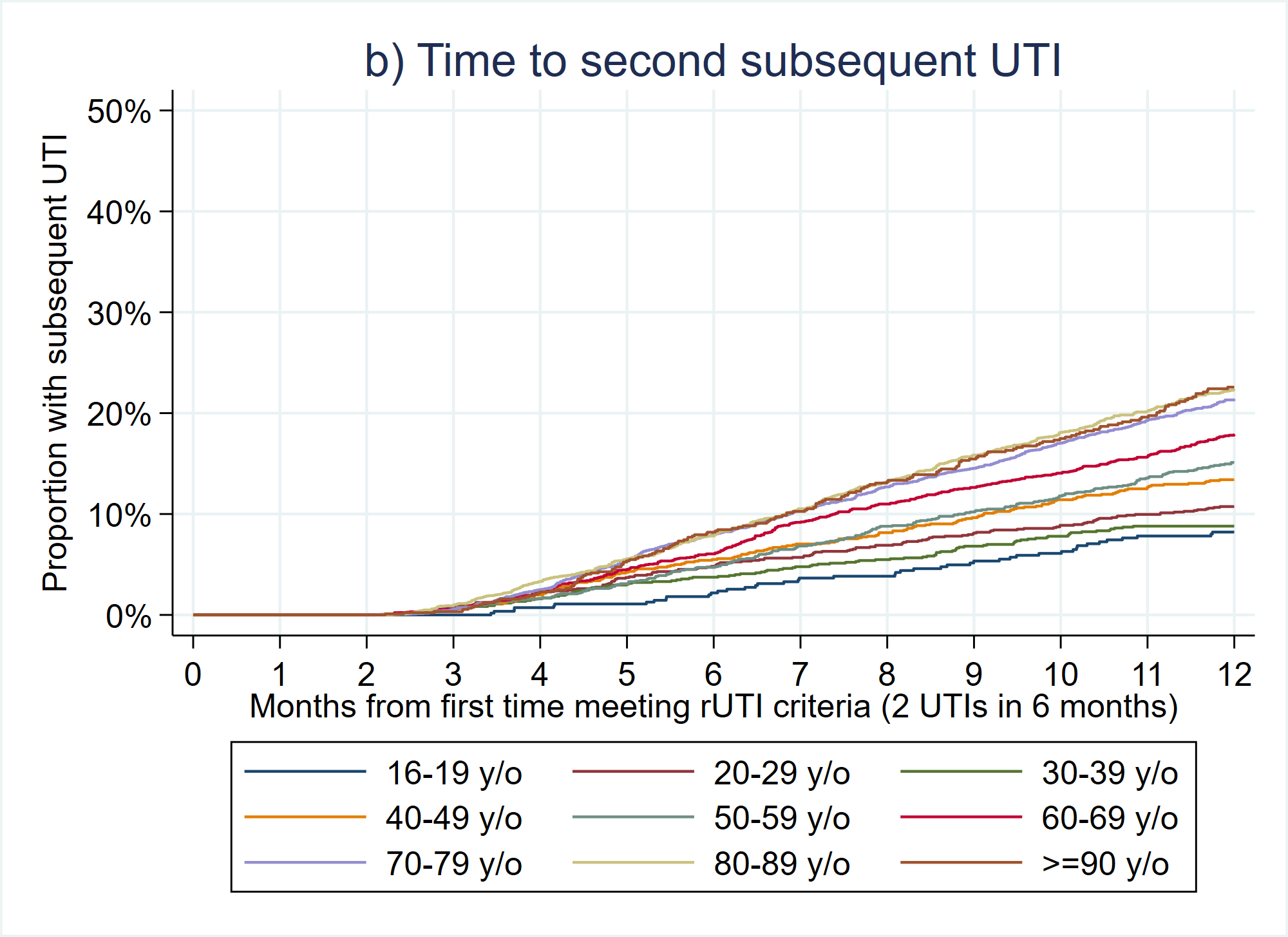 |
| 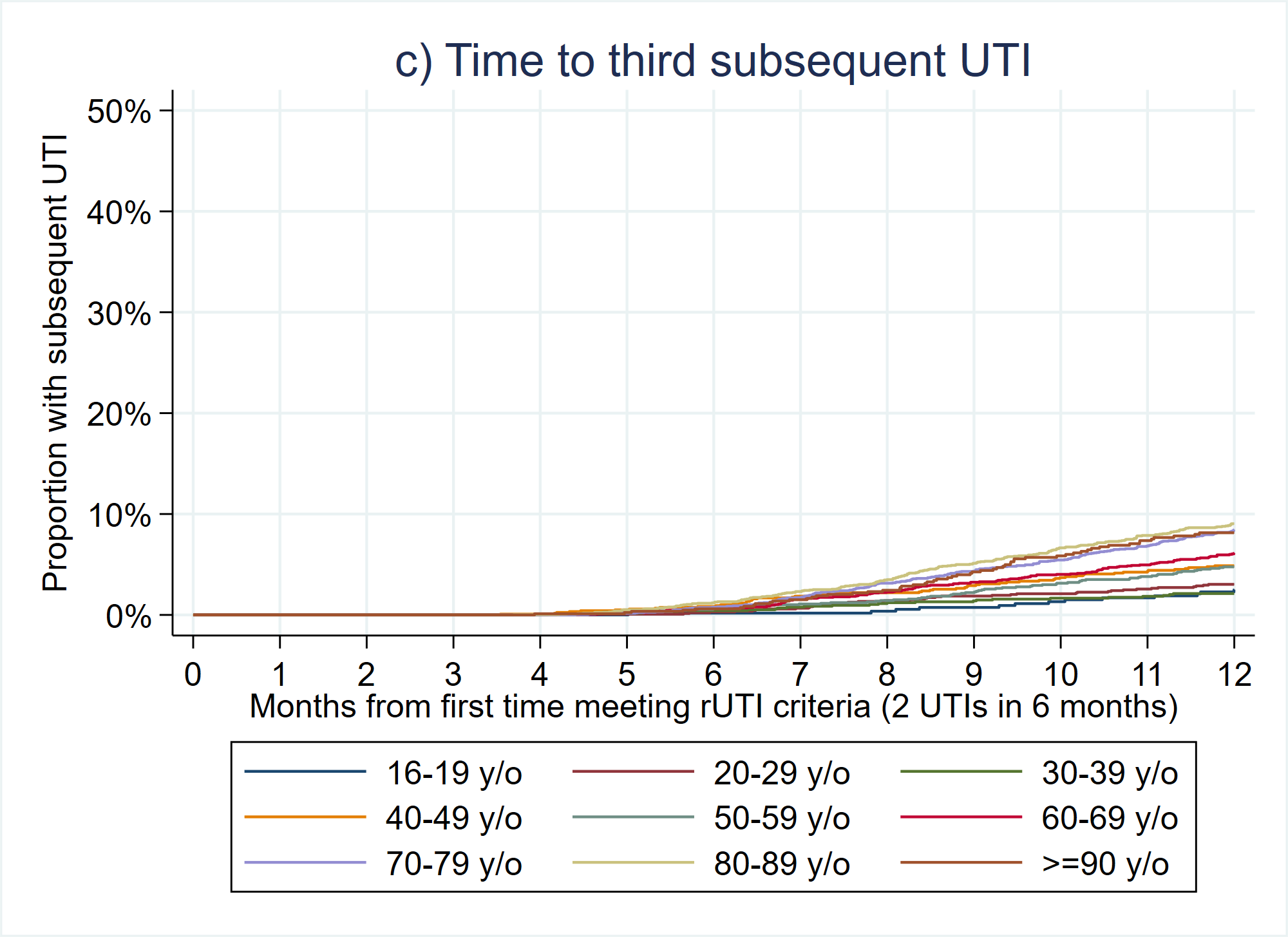 |

**TABLES**

Table S1: Microbiological result categorisation

| Bacterial species | Description |
| --- | --- |
| *E. coli* | *Escherichia coli* |
| ESBL - *E. coli* | Extended-spectrum beta-lactamase *Escherichia coli* |
| *Enterococcus* spp. |  |
| *Klebsiella* spp. |  |
| ESBL - *Klebsiella* | Extended-spectrum beta-lactamase *Klebsiella* spp. |
| *Proteus* spp. |  |
| Other | *Streptococcus agalactiae; Other Enterobacterales; Staphylococcus saprophyticus; Pseudomonas* spp.*; methicillin-susceptible Staphylococcus aureus (MSSA); methicillin-resistant Staphylococcus aureus (MRSA)*; *vancomycin-resistant* *Enterococcus* (*VRE) (incl. Enterococcus casseliflavus, faecalis, faecium, and gallinarum*) and all other species with a total frequency of less than 1000 [<0.03% of all culture results] each) |

Table S2: Distribution of UTI. Number of patients with one and with two or more UTIs during follow-up, not necessarily meeting the current rUTI definition

|  | Women with one UTI | | Women with ≥2 UTIs | | | |
| --- | --- | --- | --- | --- | --- | --- |
|  | n | % | n | % | Median UTIs per person | Interquartile range |
| 1. **Sample restricted to positive cultures in the study period (Total women=84,809)** | | | | | | |
| UTI as single positive culture* | 50,336 | 59 | 34,473 | 41 | 3 | 2-4 |
| UTI episode, 28 days or longer | 53,287 | 63 | 31,522 | 37 | 3 | 2-4 |
| UTI episode, 14 days or longer | 52,040 | 61 | 32,769 | 39 | 3 | 2-4 |
| 1. **Sample restricted to positive cultures from June 2013 (Total women=56,814)** | | | | | | |
| UTI as single positive culture* | 35,841 | 63 | 20,973 | 37 | 3 | 2-4 |
| UTI episode, 28 days or longer | 38,127 | 67 | 18,687 | 33 | 2 | 2-4 |
| UTI episode, 14 days or longer | 37,175 | 65 | 19,639 | 35 | 2 | 2-4 |
| 1. **All urine cultures in the study period (Total women=201,927)** | | | | | | |
| UTI as single positive culture* | 83,832 | 42 | 118,095 | 58 | 3 | 2-6 |
| UTI episode, 28 days or longer | 93,948 | 47 | 107,979 | 53 | 3 | 2-5 |
| UTI episode, 14 days or longer | 90,007 | 45 | 111,920 | 55 | 3 | 2-5 |
| 1. **All urine cultures from June 2013 (Total women=133,244)** | | | | | | |
| UTI as single positive culture* | 63,338 | 48 | 69,906 | 52 | 3 | 2-5 |
| UTI episode, 28 days or longer | 71,460 | 54 | 61,784 | 46 | 3 | 2-4 |
| UTI episode, 14 days or longer | 68,217 | 51 | 65,027 | 49 | 3 | 2-4 |

* UTI as single positive culture = each positive culture viewed as a separate UTI episode, regardless of the time between cultures

Table S3: Distribution of rUTI. Number of patients with one or more rUTI episodes during follow-up, according to the full rUTI definition and each of its parts

|  | Definition of new UTI episode | | | |
| --- | --- | --- | --- | --- |
|  | Event 28 days or more from the index of a previous episode | | Event 14 days or more from the index of a previous episode | |
|  | n | % | n | % |
| 1. **Only positive culture, whole study period (Total women = 84,809)** | | | | |
| Patients with two or more UTI episodes within 6 months | 15,617 | 18 | 18,129 | 21 |
| Patients with three or more UTI episodes within 12 months | 6,880 | 8 | 8,587 | 10 |
| Patients with one or more rUTI according to current definition | 15,617 | 18 | 18,129 | 21 |
| 1. **Only positive culture, data from June 2013 onwards (Total women = 56,814)** | | | | |
| Patients with two or more UTI episodes within 6 months | 10,216 | 18 | 11,881 | 21 |
| Patients with three or more UTI episodes within 12 months | 4,377 | 8 | 5,516 | 10 |
| Patients with one or more rUTI according to current definition | 10,216 | 18 | 11,881 | 21 |
| 1. **All cultures, whole study period (Total women = 201,927)** | | | | |
| Patients with two or more UTI episodes within 6 months | 64,260 | 32 | 73,682 | 36 |
| Patients with three or more UTI episodes within 12 months | 31,764 | 16 | 40,726 | 20 |
| Patients with one or more rUTI according to current definition | 64,260 | 32 | 73,682 | 36 |
| 1. **All cultures, data from June 2013 onwards (Total women = 133,244)** | | | | |
| Patients with two or more UTI episodes within 6 months | 37,930 | 28 | 44,080 | 33 |
| Patients with three or more UTI episodes within 12 months | 18,001 | 14 | 23,323 | 18 |
| Patients with one or more rUTI according to current definition | 37,930 | 28 | 44,080 | 33 |

Table S4: Baseline distribution of bacterial species. Frequencies of bacterial species at first rUTI and index UTI for women who experienced and did not experience any rUTI, overall and by age group

| Bacterial species | Age group | | | | | | | | | | | | | | | | | | | |
| --- | --- | --- | --- | --- | --- | --- | --- | --- | --- | --- | --- | --- | --- | --- | --- | --- | --- | --- | --- | --- |
|  | 16-19 | | 20-29 | | 30-39 | | 40-49 | | 50-59 | | 60-69 | | 70-79 | | 80-89 | | >=90 | | Total | |
|  | n | % | n | % | n | % | n | % | n | % | n | % | n | % | n | % | n | % | n | % |
| *E. coli* |  |  |  |  |  |  |  |  |  |  |  |  |  |  |  |  |  |  |  |  |
| rUTI | 221 | 66 | 590 | 71 | 472 | 60 | 562 | 70 | 782 | 73 | 993 | 68 | 1515 | 69 | 1315 | 65 | 471 | 68 | 6921 | 68 |
| Non-rUTI | 1748 | 68 | 4714 | 64 | 3671 | 59 | 3727 | 71 | 4172 | 70 | 4205 | 70 | 4290 | 67 | 3185 | 65 | 1203 | 63 | 30915 | 66 |
| ESBL - *E. coli* |  |  |  |  |  |  |  |  |  |  |  |  |  |  |  |  |  |  |  |  |
| rUTI | 10 | 3 | 26 | 3 | 36 | 5 | 25 | 3 | 46 | 4 | 61 | 4 | 76 | 3 | 81 | 4 | 25 | 4 | 386 | 4 |
| Non-rUTI | 66 | 3 | 212 | 3 | 149 | 2 | 151 | 3 | 175 | 3 | 186 | 3 | 196 | 3 | 152 | 3 | 55 | 3 | 1342 | 3 |
| *Enterococcus* spp. |  |  |  |  |  |  |  |  |  |  |  |  |  |  |  |  |  |  |  |  |
| rUTI | 14 | 4 | 56 | 7 | 92 | 12 | 45 | 6 | 47 | 4 | 87 | 6 | 91 | 4 | 82 | 4 | 24 | 3 | 538 | 5 |
| Non-rUTI | 105 | 4 | 626 | 8 | 881 | 14 | 377 | 7 | 377 | 6 | 331 | 6 | 397 | 6 | 329 | 7 | 87 | 5 | 3510 | 8 |
| *Klebsiella* spp. |  |  |  |  |  |  |  |  |  |  |  |  |  |  |  |  |  |  |  |  |
| rUTI | 12 | 4 | 37 | 4 | 34 | 4 | 45 | 6 | 60 | 6 | 116 | 8 | 193 | 9 | 220 | 11 | 57 | 8 | 774 | 8 |
| Non-rUTI | 56 | 2 | 200 | 3 | 202 | 3 | 184 | 3 | 266 | 4 | 326 | 5 | 452 | 7 | 377 | 8 | 169 | 9 | 2232 | 5 |
| ESBL – *Klebsiella* spp. |  |  |  |  |  |  |  |  |  |  |  |  |  |  |  |  |  |  |  |  |
| rUTI | 1 | 0 | 2 | 0 | 3 | 0.5 | 2 | 0.2 | 4 | 0.3 | 7 | 0.4 | 4 | 0.2 | 6 | 0.3 | 6 | 1 | 35 | 0 |
| Non-rUTI | 1 | 0 | 7 | 0 | 7 | 0.1 | 10 | 0.2 | 9 | 0.2 | 14 | 0.2 | 11 | 0.2 | 17 | 0.3 | 9 | 0 | 85 | 0 |
| *Proteus* |  |  |  |  |  |  |  |  |  |  |  |  |  |  |  |  |  |  |  |  |
| rUTI | 13 | 4 | 18 | 2 | 20 | 3 | 15 | 2 | 25 | 2 | 50 | 3 | 80 | 4 | 90 | 4 | 28 | 4 | 339 | 3 |
| Non-rUTI | 66 | 3 | 164 | 2 | 96 | 2 | 76 | 1 | 121 | 2 | 152 | 3 | 242 | 4 | 218 | 4 | 84 | 4 | 1219 | 3 |
| Other |  |  |  |  |  |  |  |  |  |  |  |  |  |  |  |  |  |  |  |  |
| rUTI | 48 | 15 | 111 | 13 | 130 | 17 | 112 | 14 | 104 | 10 | 180 | 12 | 252 | 11 | 209 | 10 | 77 | 11 | 1223 | 12 |
| Non-rUTI | 542 | 21 | 1473 | 20 | 1233 | 20 | 759 | 14 | 845 | 14 | 753 | 13 | 777 | 12 | 619 | 13 | 294 | 15 | 7295 | 16 |

NB: 46,598 women never experienced rUTI, non-rUTI; 10,216 women experienced ≥1 rUTI

Table S5: Multinomial logistic regression model of bacterial species on age group and recurrence group. Columns are relative risk ratios (RRR), 95% confidence interval, and p-value, with reference *E.coli* cultured in rUTI women aged 30-39.

| **Bacterial species** | **Age group** | **RRR** | **95% CI** | | **p-value** |
| --- | --- | --- | --- | --- | --- |
| ESBL - *E. coli* | 16-19 | 0.87 | 0.66 | 1.14 | 0.298 |
|  | 20-29 | 1.01 | 0.83 | 1.23 | 0.937 |
|  | 40-49 | 0.91 | 0.74 | 1.13 | 0.408 |
|  | 50-59 | 0.99 | 0.81 | 1.21 | 0.920 |
|  | 60-69 | 1.04 | 0.86 | 1.27 | 0.681 |
|  | 70-79 | 1.01 | 0.84 | 1.23 | 0.896 |
|  | 80-89 | 1.11 | 0.91 | 1.36 | 0.301 |
|  | >=90 | 1.03 | 0.79 | 1.35 | 0.822 |
|  | non-rUTI | 0.79 | 0.70 | 0.89 | 0.000 |
|  | Intercept | 0.05 | 0.05 | 0.07 | 0.000 |
| *Enterococcus* spp. | 16-19 | 0.26 | 0.21 | 0.31 | 0.000 |
|  | 20-29 | 0.55 | 0.49 | 0.61 | 0.000 |
|  | 40-49 | 0.42 | 0.37 | 0.48 | 0.000 |
|  | 50-59 | 0.37 | 0.33 | 0.42 | 0.000 |
|  | 60-69 | 0.35 | 0.31 | 0.40 | 0.000 |
|  | 70-79 | 0.37 | 0.33 | 0.42 | 0.000 |
|  | 80-89 | 0.41 | 0.36 | 0.46 | 0.000 |
|  | >=90 | 0.29 | 0.24 | 0.36 | 0.000 |
|  | non-rUTI | 1.33 | 1.21 | 1.47 | 0.000 |
|  | Intercept | 0.18 | 0.16 | 0.20 | 0.000 |
| *Klebsiella* spp. | 16-19 | 0.61 | 0.46 | 0.80 | 0.000 |
|  | 20-29 | 0.79 | 0.65 | 0.95 | 0.011 |
|  | 40-49 | 0.93 | 0.77 | 1.12 | 0.462 |
|  | 50-59 | 1.14 | 0.96 | 1.36 | 0.130 |
|  | 60-69 | 1.46 | 1.24 | 1.71 | 0.000 |
|  | 70-79 | 1.87 | 1.60 | 2.18 | 0.000 |
|  | 80-89 | 2.21 | 1.89 | 2.59 | 0.000 |
|  | >=90 | 2.27 | 1.87 | 2.75 | 0.000 |
|  | non-rUTI | 0.76 | 0.70 | 0.83 | 0.000 |
|  | Intercept | 0.07 | 0.06 | 0.08 | 0.000 |
| ESBL-*Klebsiella* spp. | 16-19 | 0.42 | 0.09 | 1.93 | 0.265 |
|  | 20-29 | 0.71 | 0.29 | 1.74 | 0.452 |
|  | 40-49 | 1.15 | 0.50 | 2.66 | 0.748 |
|  | 50-59 | 1.07 | 0.47 | 2.43 | 0.881 |
|  | 60-69 | 1.60 | 0.75 | 3.40 | 0.224 |
|  | 70-79 | 0.99 | 0.44 | 2.22 | 0.983 |
|  | 80-89 | 1.93 | 0.91 | 4.08 | 0.086 |
|  | >=90 | 3.43 | 1.53 | 7.67 | 0.003 |
|  | non-rUTI | 0.62 | 0.42 | 0.93 | 0.021 |
|  | Intercept | 0.00 | 0.00 | 0.01 | 0.000 |
| *Proteus* spp. | 16-19 | 1.43 | 1.07 | 1.92 | 0.015 |
|  | 20-29 | 1.23 | 0.97 | 1.55 | 0.090 |
|  | 40-49 | 0.76 | 0.57 | 1.00 | 0.049 |
|  | 50-59 | 1.05 | 0.82 | 1.34 | 0.701 |
|  | 60-69 | 1.38 | 1.09 | 1.74 | 0.007 |
|  | 70-79 | 1.96 | 1.58 | 2.43 | 0.000 |
|  | 80-89 | 2.41 | 1.94 | 3.00 | 0.000 |
|  | >=90 | 2.36 | 1.81 | 3.08 | 0.000 |
|  | non-rUTI | 0.93 | 0.82 | 1.05 | 0.241 |
|  | Intercept | 0.03 | 0.02 | 0.04 | 0.000 |
| Other | 16-19 | 0.91 | 0.81 | 1.02 | 0.095 |
|  | 20-29 | 0.91 | 0.83 | 0.98 | 0.019 |
|  | 40-49 | 0.62 | 0.56 | 0.68 | 0.000 |
|  | 50-59 | 0.59 | 0.53 | 0.64 | 0.000 |
|  | 60-69 | 0.55 | 0.51 | 0.61 | 0.000 |
|  | 70-79 | 0.55 | 0.51 | 0.61 | 0.000 |
|  | 80-89 | 0.58 | 0.53 | 0.64 | 0.000 |
|  | >=90 | 0.69 | 0.61 | 0.79 | 0.000 |
|  | non-rUTI | 1.23 | 1.15 | 1.32 | 0.000 |
|  | Intercept | 0.27 | 0.25 | 0.30 | 0.000 |

RRR = Relative risk ratio

Table S6: Bacterial species in first and second UTI events within a rUTI episode, across participants

| Bacterial species in the first UTI of distinct rUTI events | Bacterial species in the second UTI event (where the rUTI definition was met) of each distinct rUTI episode | | | | | | | | | | | | | | | |
| --- | --- | --- | --- | --- | --- | --- | --- | --- | --- | --- | --- | --- | --- | --- | --- | --- |
|  | *E.Coli* | | *ESBL - E.Coli -* | | *Enterococcus* spp. | | *Klebsiella* spp. | | *ESBL -Klebsiella* spp. | | *Proteus* spp. | | Other | | Total | |
|  | n | % | n | % | n | % | n | % | n | % | n | % | n | % | n | % |
| *E. coli* | 6,813 | 81 | 172 | 2 | 289 | 3 | 333 | 4 | 11 | 0.1 | 151 | 2 | 608 | 7 | 8,377 | 100 |
| ESBL – *E. coli* | 183 | 38 | 226 | 47 | 16 | 3 | 16 | 3 | 2 | 0.4 | 14 | 3 | 29 | 6 | 486 | 100 |
| *Enterococcus* spp. | 221 | 35 | 16 | 3 | 251 | 39 | 43 | 7 | 2 | 0.3 | 17 | 3 | 90 | 14 | 640 | 100 |
| *Klebsiella* spp. | 293 | 29 | 15 | 2 | 43 | 4 | 494 | 49 | 15 | 2 | 22 | 2 | 117 | 12 | 999 | 100 |
| ESBL - *Klebsiella* spp. | 6 | 14 | 1 | 2 | 1 | 2 | 4 | 9 | 28 | 64 | 1 | 2 | 3 | 7 | 44 | 100 |
| *Proteus* spp. | 142 | 33 | 12 | 3 | 18 | 4 | 41 | 10 | 2 | 1 | 156 | 37 | 55 | 13 | 426 | 100 |
| Other | 473 | 32 | 36 | 2 | 100 | 7 | 106 | 7 | 7 | 1 | 40 | 3 | 721 | 49 | 1483 | 100 |
| Total | 8,131 | 65 | 478 | 4 | 718 | 6 | 1,037 | 8 | 67 | 1 | 401 | 3 | 1,623 | 13 | 12,455 | 100 |

Table S7: Species patterns in pairs of consecutive UTI episodes within participants with 2+ UTIs who never met the rUTI definition

| Bacterial species in the index UTI | Bacterial species in the second UTI episode | | | | | | | | | | | | | | | |
| --- | --- | --- | --- | --- | --- | --- | --- | --- | --- | --- | --- | --- | --- | --- | --- | --- |
|  | *E.Coli* | | *ESBL - E.Coli -* | | *Enterococcus* spp. | | *Klebsiella* spp. | | *ESBL -Klebsiella* spp. | | *Proteus* spp. | | Other | | Total | |
|  | n | % | n | % | n | % | n | % | n | % | n | % | n | % | n | % |
| *E. coli* | 5,882 | 77 | 197 | 3 | 345 | 5 | 367 | 5 | 9 | 0.1 | 182 | 2 | 691 | 9 | 7,673 | 100 |
| ESBL – *E. coli* | 182 | 55 | 67 | 20 | 19 | 6 | 22 | 7 | 3 | 1 | 12 | 4 | 28 | 8 | 333 | 100 |
| *Enterococcus* spp. | 257 | 47 | 16 | 3 | 132 | 24 | 29 | 5 | 1 | 0.2 | 19 | 3 | 93 | 17 | 547 | 100 |
| *Klebsiella* spp. | 350 | 52 | 10 | 1 | 33 | 5 | 180 | 27 | 3 | 0.4 | 25 | 4 | 76 | 11 | 677 | 100 |
| ESBL - *Klebsiella* spp. | 14 | 58 | 0 | 0 | 2 | 8 | 3 | 13 | 2 | 8 | 1 | 4 | 2 | 8 | 24 | 100 |
| *Proteus* spp. | 161 | 55 | 7 | 2 | 13 | 4 | 19 | 7 | 1 | 0.3 | 58 | 20 | 33 | 11 | 292 | 100 |
| Other | 660 | 52 | 33 | 3 | 81 | 6 | 76 | 6 | 5 | 0.4 | 37 | 3 | 386 | 30 | 1,278 | 100 |
| Total | 7,506 | 69 | 330 | 3 | 625 | 5 | 696 | 6 | 24 | 0.2 | 334 | 3 | 1,309 | 12 | 10,824 | 100 |

Table S8: Intrinsic antibiotic resistance for the pathogen-drug combinations considered in this study, based on EUCAST guidance

|  | CIP | AMC | NIT | TRIM | AMOX | CEF | FOS | PIV |
| --- | --- | --- | --- | --- | --- | --- | --- | --- |
| *E. coli* | 0 | 0 | 0 | 0 | 0 | 0 | 0 | 0 |
| *Enterococcus faecalis* | 0 | 0 | 0 | 1 | 0 | 1 | 0 | 1 |
| *Enterococcus faecium* | 0 | 1 | 0 | 1 | 1 | 1 | 0 | 1 |
| *Klebsiella spp.* | 0 | 0 | 0 | 0 | 1 | 0 | 0 | 0 |
| *Proteus* | 0 | 0 | 1 | 0 | 0 | 0 | 0 | 0 |

1 = intrinsic, 0 = not intrinsic, CIP = Ciprofloxacin, AMC = co-amoxiclav, NIT = nitrofurantoin, TRIM = trimethoprim, AMOX = amoxicillin, CEF = cefalexin, FOS = fosfomycin, PIV = pivmecillinam
